# Ewing's Sarcoma and Second Malignancies

**DOI:** 10.1155/2011/736841

**Published:** 2010-10-13

**Authors:** Joshua D. Schiffman, Jennifer Wright

**Affiliations:** ^1^Division of Pediatric Hematology/Oncology, University of Utah School of Medicine, Salt Lake City, UT 84108, USA; ^2^Center for Children's Cancer Research, Huntsman Cancer Institute, Salt Lake City, UT 84112, USA

## Abstract

Ewing's sarcoma (ES) is a rare tumor that is most common in children and young adults. Late effects of ES therapy include second cancers, a tragic outcome for survivors of such a young age. This paper will explore the frequencies and types of malignancies that occur after ES. Additionally, it will review how second malignancies have changed with the shift in treatment from high-dose radiation to chemotherapy regimens including alkylators and epipodophyllotoxins. The risk of additional cancers in ES survivors will also be compared to survivors of other childhood cancers. Finally, the possible genetic contribution to ES and second malignancies will be discussed.

## 1. Introduction

The 5-year survival rate for Ewing's sarcoma/Ewing's sarcoma family of tumors (ES) has increased from <20% prior to the use of systemic therapy in the early 1970's [1] to around 70% with current therapy in localized disease [2]. This improvement in survival is credited to improved surgical techniques, refinements in radiation therapy, and intensified multiagent chemotherapy. In ES as well as in all childhood cancers, late effects are being discovered as survival rates improve and the survivor pool enlarges. Subsequent malignant neoplasms (SMN) have been recognized as a particularly tragic late effect of childhood cancer therapy. Radiation therapy (RT), anthracyclines, alkylators, and epipodophyllotoxins are all used in modern ES therapy and have been associated with SMN. This paper will review the rates of and common types of SMN after ES therapy, the changes in SMN as ES therapy has changed over time, and risk of SMN in ES survivors compared to survivors of other childhood cancers.

## 2. Incidence of Second Malignancies after Ewing's Sarcoma

### 2.1. Radiation Treatment Era

The original reports of SMN after ES therapy were most notable for high rates of secondary bone cancers. The Late Effects Study Group first reported on SMN in ES and osteosarcoma (OS) survivors together. They found a SMN annual incidence of 148.5 per 100,000 per year in the 1,066 bone tumor survivors [3]. Patients included in this report were treated from 1950 through 1970. No hematologic SMN were reported, but 62.5% of the SMN were bone or soft tissue sarcomas (STS). Other SMN included one each of breast cancer, brain tumor, and nondisclosed cancer type. This high rate of secondary sarcomas would be expected in an ES treatment era when many patients would have been treated with high-dose RT only. The grouping of ES and OS together, however, makes it difficult to determine a true risk of SMN for ES survivors given that the cohort may include OS patients with a high genetic risk for SMN (e.g. germline *p*53 mutations/ Li-Fraumeni Syndrome(LFS)). An updated report from the same group in 1987 looked specifically at secondary bone cancers by primary cancer type. Patients with a primary ES had a relative risk (RR) of 649 and an absolute excess risk (AER) of 59.6 per 100,000 per year for secondary bone tumors. It was also noted that the risk of secondary bone tumors (not specific to ES primary) was highest in those who received >60 Gy [4]. 

Six of 24 long-term survivors treated at MD Anderson in the 1970's developed SMN in the RT field; four of these were OS [5]. A brief report of early Surveillance, Epidemiology, and End Results Program (SEER) data found a cumulative SMN incidence of 2.2% in those who had survived ES for greater than 5 years, with an observed/expected (O/E) ratio of 8.5 [6]. The registry included patients treated in the 1980's, and while the majority of SMN were various solid tumors, a small number of secondary myeloid leukemias were noted. A small study of 76 ES patients from Iowa looked at the incidence of late effects by local control modality. All 3 patients that developed SMN had RT only for local control. SMN included one each of OS, cervical cancer, and breast cancer [7]. Kuttesch Jr. et al. reviewed 266 ES survivors treated prior to 1990 from 3 institutions with a median followup of 9.5 years found 16 SMN, 14 of which were solid tumors (10 sarcomas, 3 carcinomas, and one meningioma). Risk of SMN was higher in those who received RT compared to those who did not, and the risk of secondary sarcoma was higher with higher RT dose. The reported cumulative rate of SMN was 9.2% at 20 years [8]. In summary, the above studies point out the high risk of secondary malignancies (particularly sarcomas within the radiation field) for ES patients who receive radiation. Although they include small numbers of patients, the reports note a trend of increasing risk of SMN with increasing radiation dose.

### 2.2. Chemotherapy Treatment Era Prior to Standard High-Dose Alkylators and Epipodophyllotoxins

More detailed reports focused solely on SMN in survivors of ES have come forth since the original limited studies above. The largest of these studies are summarized in Table 1. In the Kuttesch Jr et al. review that was described above, only 7.9% of patients did not receive radiation. Most patients received cyclophosphamide (97%) and doxorubicin (86%) at median doses of 10,900 mg/m^2^ and 394 mg/m^2^, respectively. Most patients did not receive etoposide (18%) or ifosfamide (15%). Only 2 of the 16 SMN were hematologic, one each of lymphoid and myeloid leukemias [8]. 

A very long-term followup of patients treated at the Mayo Clinic between 1960 and 1980 revealed a 7.3% overall rate of SMN in a group of patients whose overall survival rate was just 22%. The majority of patients received vincristine, dactinomycin, cyclophosphamide, and doxorubicin (VACA). SMN included one sarcoma and two carcinomas. All patients with SMN had received RT, however the SMN occurred in the RT field in only one of these three patients [9]. 

McLean et al. reviewed the Dana Farber Cancer Institute experience between 1971 and 1988 at a median 10.2 year survival in 82 patients treated for ES. Seventy-five (91.5%) patients received vincristine, actinomycin, and cyclophosphamide (VAC) or VACA. Seven patients developed SMN at a median latency of 14.4 years from primary diagnosis. Four sarcomas, one carcinoma, and 2 hematologic SMN were noted. The cumulative risk of SMN was 6.7% and 42.8% at 10 and 20 years, respectively. Although primary therapy did not include ifosfamide and etoposide (IE), the single patient who developed secondary acute myeloid leukemia (AML) had received these drugs for recurrent ES. No difference in risk was found based on radiation dose or doxorubicin administration [10].

As chemotherapy protocols for ES started to include higher doses of alkylators, but still prior to the routine use of etoposide, the ratio of solid to hematologic SMN began to decrease. A review of the German experience from 1981–1991 included a majority of patients treated with VACA with some high-risk patients treated with vincristine, actinomycin, ifosfamide, and doxorubicin (VAIA). They reported an overall rate of SMN of 1.2% after ES with a relatively short median followup of 5.1 years. No treatment factors were found to be statistically significant in predicting the development of SMN, although there was a trend toward more solid tumor SMN in those who were treated with RT. The three solid tumor SMN (all sarcomas) were diagnosed at 6.8, 9.8, and 11.4 years after ES treatment, while all but one of the five hematologic SMN were diagnosed within 3 years of ES treatment [11].

The Rizzoli Institute in Italy first reported on their SMN findings after ES therapy in 1989. At that time, they found 4 out of 144 localized ES survivors developed 3 radiation induced sarcomas and one acute lymphoblastic leukemia (ALL) [12]. Their more recent review of 597 ES patients found 14 SMN, 11 of which were solid tumors. Similar to other reports, they found the risk for SMN to be higher with higher doses of radiation. The mean followup of patients who developed a hematologic SMN was shorter than those who developed a solid tumor SMN. The estimated rate of any SMN at 5, 10, and 20 years after original diagnosis was 3%, 6.5%, and 12.7% respectively [13]. When analyzed by chemotherapy regimen, the SMN rate was 5.9% after vincristine, doxorubicin, and cyclophosphamide (VDC), 1.8% after VACA, and 1.7% after VACA plus IE with median followup for each group of 7.6, 7.4, and 4.1 years; respectively.

The Childhood Cancer Survivor Study (CCSS) is a well-described multiinstitutional cohort of pediatric cancer patients treated between 1970 and 1986 with a minimum 5-year survival. Information for the CCSS was gained via self-report questionnaires, but SMN were confirmed through medical record review when reported. Cervical dysplasia, nonmelanoma skin cancer (NMSC), meningiomas, and SMN that occurred prior to inclusion (i.e., less than 5 years from the time of primary cancer diagnosis) were specifically excluded. The CCSS recently reported long-term outcomes of the ES patients in their cohort and found 36 SMN in 34 of the 403 patients, or a cumulative incidence of 9% [14]. The median time from first diagnosis to SMN was 14.5 years. Of the 34 patients who developed SMN, 26 (86.7%) received radiation. The SIR in all survivors, those who received radiation, and those who did not receive radiation, was 5.9, 6.6, and 3.3, respectively. Notably, 11 women developed 13 breast cancers, 7 of which were in the radiation field. The SIR for subsequent breast cancer after whole-lung radiation (1200–1500 cGy) was 36.0. Only 2 hematologic SMN were reported.

These six studies demonstrate that as chemotherapy became standard in ES therapy, but before the routine use of IE, hematologic SMN became more common. Solid tumor SMN continued to occur, still most commonly after RT exposure.

### 2.3. Chemotherapy Treatment Era: High Dose Alkylators and Epipodophyllotoxins

The distinction of patients who received high-dose alkylators and epipodophyllotoxins is important due to the distinct risk of secondary AML/myelodysplastic syndrome (MDS) that follows exposure to these agents. In particular, cyclophosphamide has been linked to monosomy 5 and monosomy 7 associated MDS with a latency of 5 years or more, and etoposide has been linked to 11q23 associated AML with a latency of 2 years or less [15–17].

The first American cooperative group trial (combined Children's Cancer Group/Pediatric Oncology Group (CCG/POG) INT-0091) to add IE to standard frontline therapy accrued patients from 1988 to 1992. This study randomized patients to VACA (Regimen A) or VACA alternating with IE (Regimen B) with stratification for the presence of metastases. Due to excellent accrual, a third arm was added. This arm (Regimen C) assigned patients with metastatic disease to VDC alternating with IE, but with cumulative doses of ifosfamide 140 g/m^2^, cyclophosphamide 17.6 g/m^2^, and doxorubicin 450 mg/m^2^ (compared to 90 g/m^2^, 9.6 g/m^2^, and 375 mg/m^2^, resp., on Regimen B). Seven of 398 participants without metastatic disease developed a nSMN. Four SMN were hematologic and 3 SMN were solid tumors (2 sarcomas and one ovarian tumor). No differences were found in SMN rates for the standard (Regimen A) versus experimental (Regimen B) arms, however, it is not reported if hematologic SMN were more common in the experimental arm [2]. When patients with localized and metastatic disease were evaluated, twelve patients developed a hematologic SMN at a median of 8.03 years and only one additional solid tumor SMN was found in addition to the prior report [18]. Six of the additional 8 patients with hematologic SMN received therapy per Regimen C. In a multivariate analysis of the patients with secondary AML or MDS, only treatment Regimen C was associated with increased risk. The overall rate of secondary AML/MDS was 2% at 5 years, but was 11% in those treated on Regimen C. Patients were analyzed in regard to granulocyte colony stimulating factor (G-CSF) exposure, and no dose relation risk was noted for the development of AML/MDS [18]. A larger review of recent CCG/POG legacy ES protocols, which included the above study, found 9 solid tumor SMN in 1,156 patients. Cumulative incidence for solid tumor SMN after ES was 1.8% at 10 years. This paper also evaluated SMN after OS, and the solid tumor SMN risk in the combined ES/OS group was increased both in patients who received RT as well as those exposed to etoposide and cyclophosphamide, all of which are more likely in ES therapy than OS therapy [19]. 

The European Intergroup Cooperative Ewing's Sarcoma Study 92 (EICESS-92) enrolled 690 evaluable patients with ES from 1992 to 1999. Standard risk patients were randomized to VAIA alone or VAIA for four courses then completion of therapy with VACA and high-risk patients was randomized to VAIA or etoposide plus VAIA. Six SMN were reported for a total incidence of 0.93%, at the short median followup of 56 months. There was one secondary sarcoma, one secondary carcinoma, two cases of ALL, and two cases of AML/MDS, both with monosomy 7. There was a trend toward increased risk of SMN in patients exposed to etoposide. Standardized incidence ratios (SIRs) for the hematologic SMN were calculated and were 33.4 and 30.9 compared to SEER and the Saarland Registry populations, respectively [20]. The strikingly low rate of SMN in this study (predominantly composed of hematologic SMN) may be due to median followup of less than five years, as solid tumor SMN have been shown to have a longer latency than hematologic SMN. 

A Memorial Sloan Kettering Cancer Center protocol utilized for sarcomas in the 1990's was reviewed for risk of SMN. All patients received 16,800 mg/m^2^ of cyclophosphamide, 300 mg/m^2^ of doxorubicin, 27,000 mg/m^2^ of ifosfamide, and 1,500 mg/m^2^ of etoposide. Most patients had ES, but some patients with desmoplastic small round-cell tumor and other sarcomas were included. Five of 86 patients (5.8%) developed AML by the median followup of 24 months. The 40 month cumulative incidence of AML was 8% [21]. Of note, the cumulative doses of both ifosfamide and etoposide used in this protocol were lower than the current Children's Oncology Group (COG) standard for ES. 

A report from St Jude's Children's Research Hospital records revealed 12 SMN, 8 hematologic, and 4 solid tumors, in 237 patients treated for ES. This was a cumulative incidence of 5.1%, with 5 and 10 year rates of 3% and 4.7%, respectively. When compared to SEER cancer rates, this was a SIR of 17.8 with AER of 5.4%. All but one of the eight different protocols examined included IE, with the ifosfamide dose for the 3 most recent protocols being 33% higher than in earlier regimens. The risk for SMN was noted to be higher in those treated on more recent protocols and in those with a lower stage at diagnosis, presumably due to increased survival and thus increased followup time [22]. 

These six reports, in which the vast majority of patients were exposed to high-dose alkylators and/or epipodophyllotoxins, highlight the increase of hematologic SMN (especially myeloid) at a short latency after therapy.

### 2.4. Mixed Treatment Era

The following studies are notable for their lack of description of treatment specifics and/or the inclusion of patients treated in different treatment eras to allow for the inclusion of large numbers of patients. The previously reviewed Mayo Clinic followup was updated to include patients treated in more recent years (1975–1999) and found 29 SMN in 397 ES patients [23]. This correlates to a SMN rate of 6.5%, including hematologic SMN, at a much shorter followup than the prior report from this group. Chemotherapy regimens were not specifically described in this report. 

A single institution Dutch review of patients with extraosseous ES treated from 1979–2008 found a single patient out of 16 that had both a second and third cancer (breast tumors with different histologies) in the local radiation field. No specifics of chemotherapy regimens were noted, other than the use of both soft tissue sarcoma and ES protocols. Mean followup was 8.4 years [24]. A Turkish report of a minimum 3 year followup of childhood cancer survivors reported two of 30 (6.6%) ES patients had developed SMN [25]. This study is most notable for a very high lost to followup rate of 65%, which may vastly underestimate SMN risk. SMN were reported at a rate of 7% at a mean of 13.1 years in ES survivors treated at the National Cancer Institute from 1965–1992 [26].

ES patients who received stem-cell transplant for metastatic, refractory, or recurrent disease on the high risk EICESS protocol from 1986–1994 were analyzed for SMN. Of the 36 patients who underwent transplant, 26 were autologous and 10 were allogeneic. While chemotherapy regimens varied, all patients received total body irradiation as part of their preparatory regimen. Median followup was 7.4 years from diagnosis and 6.7 years from transplant. There were 2 cases of secondary MDS and 1 case of liposarcoma, leading to a cumulative incidence of 8.3% in a population in which 50% of patients died of disease [27]. 

The most recent and largest report of second malignancies in a cohort of ES patients is from the SEER data. The SEER registries collect cancer diagnosis and treatment information from various geographic regions of the United States, covering about 10% of the country's population [28]. Sultan et al. reviewed patients in the SEER database that were diagnosed with ES between 1973 and 2005. Of 1,166 patients with ES, 35 developed a SMN. SMN was more common in patients treated in an earlier time period (1973–1985) and with RT compared to patients who did not develop SMN. There was no difference in rates of radiation between those who developed solid tumors versus hematologic malignancies. Hematologic malignancies were more common in the later time periods (1985–2005), consistent with the standard use of etoposide during these periods. Hematologic SMN occurred after a shorter latency from diagnosis (36 months versus 98 months for solid tumors), as noted in many of the above reports. The estimated risks of SMN at 5, 10, and 20 years after original diagnosis were 2.1%, 4.4%, and 8%, respectively. The risk of SMN was higher in patients diagnosed with ES before the age of 20 years. Two of these patients developed third malignancies [29].

## 3. Types of Second Malignancies after Ewing's Sarcoma

The most common solid tumor SMN after ES is OS, followed by malignant fibrous histiocytoma (MFH) [8, 13, 23, 30, 31]. OS has been noted to comprise about 50–60% [8, 10, 31, 32] of solid tumor SMN, although OS has made up a smaller percentage after therapy in more recent eras [19]. Cases of neuroblastoma [14, 29], neuroepithelioma [29], teratocarcinoma [29], germinoma [29], endometrial sarcoma [29], liposarcoma [20, 23, 27], spindle cell sarcoma [9], dermatofibrosarcoma [29], fibroscarcoma [8, 23], undifferentiated sarcoma [19], malignant melanoma [13, 29], anaplastic astrocytoma [19], breast cancer [23, 24, 31, 33], clear cell adenocarcinoma [29], papillary thyroid cancer [19, 22, 23, 34], renal cell carcinoma [19], malignant thymoma [6], palatal [10] and parotid [35] mucoepidermoid carcinoma, colon cancer [6], gastric adenocarcinoma [10], bronchioalveolar carcinoma [8, 13], small cell lung cancer [13], squamous cell carcinoma of the cervix [22], and squamous cell carcinoma of shoulder [20] have all been reported after treatment for ES. Several of these solid tumor SMN occurred far from the radiation field or in patients who received no RT [7, 34, 35]. Second benign neoplasms have also been reported after ES, including prolactinoma [23], uterine leiomyoma [23], breast fibroadenoma [23], thyroid adenoma [23], basal cell carcinoma [8], and meningioma [8], but due to different methods of reporting their overall rates are unknown. Carcinoma in situ of the cervix [8, 23] has been noted in ES patients, some of whom had pelvic or vertebral RT, but it is unclear if these occur at rates different from in the general population. Fuchs et al. noted no difference in latency periods in the onset of secondary sarcomas compared to carcinomas [23].

The most common hematologic SMN in all reports is AML/MDS, which comprises about 60% of cases [18, 27, 29]. Other hematologic SMN include both B-cell and T-cell lineage ALL [8, 12, 18, 20] Hodgkin lymphoma [10, 29], non-Hodgkin lymphoma [29, 36], and multiple myeloma [29]. 

Many of those who reported on outcomes after SMN note better survival for those with solid tumors than for those with hematologic SMN [11, 22, 29]. Fuchs et al. report poor outcomes for secondary hematologic cancers and sarcomas, with better outcomes in secondary carcinomas [23].

## 4. Risk of Second Malignancies after Ewing's Compared to Other Childhood Cancers

The CCSS has reported the largest study comparing SMN after a variety of primary pediatric cancers. While not all cancer types were included in the cohort, the most common pediatric cancers, including bone tumors, are represented. At a median followup of 15.4 years, 314 SMN were reported in the 13,581 CCSS cohort members. The cumulative rate of SMN was 3.2% at 20 years after original diagnosis. The SIR of the entire cohort was 6.38 when compared to SEER. This particular CCSS report did not evaluate patients with ES separately, but rather they are included in the “bone tumor” or “soft-tissue sarcoma” primary malignancy groups. SIR for bone tumor primary was 4.5 with overall incidence of 2.05% at 20 years. There were no secondary leukemias in patients with primary bone tumors and the overall rate of SMN was 8.4% [37]. The probable reasons for the lack of hematologic SMN in the bone cancer survivors in this study include the treatment era of the cohort (prior to the standard use of epipodophyllotoxins and high-dose alkylators) and the exclusion of SMN that developed prior to 5 years from original diagnosis, as noted previously.

A report of British survivors suggests that the risk of bone tumors as SMN is higher after ES than other childhood cancers, with a relative risk (RR) in ES of 267, secondary only to those with heritable retinoblastoma (RR of 381) and with all childhood cancer types with an RR of 43 [32]. Inskip and Curtis' review of SEER data found that ES survivors' risk for any SMN (EAR 40.5) was second only to those with primary Hodgkin lymphoma (EAR 43.2) [38]. One review of secondary STS noted the highest risk was in those whose primary tumor was ES, who had a 7.2 times risk compared to other diagnoses. The SIR for STS after ES was 327, with the next closest being Hodgkin lymphoma and primary STS with SIRs of 73 and 75, respectively [39].

The CCSS noted that secondary breast cancers in women not exposed to chest RT are most common in bone and STS primaries. SIRs for secondary breast cancers after RT for Hodgkin lymphoma were 26.3 and bone sarcomas were 19.4 [33]. No rate was specifically noted for ES survivors, again making it difficult to know if genetically predisposed patients such as those with LFS are included in this rate. 

Long-term followup of Dutch childhood cancer survivors treated between 1966 and 1996 revealed a mortality rate from SMN of 1.4% at 16.1 years [40], without disease-specific rates reported. A review of the Nordic cancer registry found a cumulative incidence of 1.07% of SMN in pediatric patients diagnosed with a childhood cancer from 1960–1987, with the rate reaching 3.5% in 25-year survivors. There was a higher RR of SMN in those exposed to RT, and patients with lymphomas made up 25% of those who developed SMN [41].

## 5. Genetic Contribution to Ewing's Sarcoma and Secondary Malignancies

One of the distinguishing features of ES is the predominance of the (11; 22) (q24;  q12) chromosomal translocation that encodes the EWS/FLI-1 oncoprotein. Many recent molecular advances have been made in understanding the pathogenesis of ES [42] and these other involved pathways may offer an informative perspective to explain the high incidence of secondary malignancies seen in ES (and the risk of ES as SMN). Alterations in the retinoblastoma (RB) pathway are thought to be cooperating mutations in ES [43], and ES occurring as SMN after treatment for heritable RB continue to be described in the literature [44–49]. Perhaps the underlying germline RB mutations found in patients with heritable RB combine with acquired EWS/FLI-1 translocations in mesenchymal or neuroectodermal tissues to create the necessary alterations to develop ES as SMN in RB patients. 

Interestingly, no known hereditary cancer syndromes have ever been associated with ES (other than the RB connection described above). However, the possibility of ES predisposition genes have been discussed based on epidemiological evidence [50]. In particular, the repeated association between ES and hernias [51], the association between ES and increased paternal age [52], sibling reports [53, 54], and the repeated observation that ES almost exclusively occurs in Caucasians [55] suggest some type of genetic contribution to risk of ES. One could speculate that this same genetic risk, even if only modest, contributes to the observed increased risk of SMN after treatment for ES compared to other childhood cancers. As more research is done on the genetic epidemiology of ES, a better understanding of increased SMN risk in ES may become clear.

## 6. Discussion

The incidence of SMN after ES treatment has varied in many reports. Much of this variation is due to the changing therapies throughout different treatment eras. The frequency of RT in all childhood cancer therapy has decreased from 56% in 1973–79 to 28% in 1995–2002 [38], and this trend has been followed in ES treatment protocols. Although solid tumor SMN have decreased in frequency, many reports note significantly increased risk in patients who received RT or with increased RT dose. Hematologic SMN have increased with intensified chemotherapy regimens containing high-dose alkylators and epipodophyllotoxins, which are known to cause secondary leukemias. Since hematologic SMN generally have a shorter latency period than solid tumor SMN, solid tumor SMN are likely to be underrepresented in studies with short followup. Based on the reviews of ES patients treated with current therapies, a likely estimate of all SMN would be 5-6% at 10 years after diagnosis. 

Intensification of chemotherapy for ES has in part been possible due to the use of G-CSF, but little information is available regarding G-CSF's potential role in hematologic SMN. One study in ALL patients found secondary AML rates were slightly increased in those who received G-CSF compared to those who did not [56]. An analysis of AML patients found a higher risk of relapse in those treated with G-CSF who had increased quantities of G-CSF receptor (G-CSFR) isoform IV in blasts at diagnosis [57]. This suggests that there may be a subgroup of patients whose tumor biology puts them at excess risk for malignancy when etoposide and G-CSF are used in combination. While it is not clear that such a relationship exists in ES patients, DNA damage to hematopoeitic stem cells caused by intensified chemotherapy may create leukemic blasts which are then perpetuated by G-CSF leading to the high rate of leukemia observed as ES-related SMN.

Many comparative studies have found that ES survivors have a higher risk of SMN compared to survivors of other childhood cancers. While much of this risk can be attributed to therapeutic modalities, there are also higher than expected rates of SMN not clearly related to treatment. As discussed above, ES is not clearly a part of any cancer predisposition syndrome. However, ES tumors have been shown to contain alterations in RB and *p*53 [58], which are known to be mutated in hereditary RB and LFS, respectively. Considered with the epidemiology data that suggests a mild to modest genetic contribution to ES risk, the high rate of SMN after ES raises the question if the underlying biology of ES patients contributes to this excess SMN risk.

This paper is limited by the available data. The cited reports used various statistical methods making it difficult to compare actual outcomes. Single cases may be reported more than once, especially with the use of large population databases such as SEER. Followup of SEER patients is limited in that an SMN will not be reported if the patient has moved out of the catchment area since the primary diagnosis. In some studies, most notably the CCSS cases would be excluded if they developed SMN before surviving 5 years from original diagnosis, leading to an underestimation of SMN risk. None of the reports evaluated outcomes by primary tumor site, which may or may not be similar to outcomes by local control modality. Nevertheless, from this paper one can conclude that ES survivors are at significant risk for SMN, particularly sarcomas and other solid tumors in RT fields and for myeloid SMN after epipodophyllotoxin and alkylator therapy. Additionally, but likely related in part to ES therapy, the risk in these patients may be higher than in those treated for other childhood cancers. ES survivors should be educated on the risk of SMN and followed closely throughout their lifetime.

## Figures and Tables

**Table 1:** 

Author (population studied)	Median Followup	Treatment years	Total patient (#)	SMN (#)	SMN types	Significant Risks for SMN	5/10/15/20 year SMN rates	Risk calculation
Kuttesch, et al. (NCI, SJCRH, Florida)	9.5 years	Prior to 1990	266	16	14 solid tumor 2 hematologic	Treating institution, Any RT, Higher sarcoma risk with higher RT (≥60 Gray) dose	NR 5% NR 9.2%	AR = 33.8/10 K PY for sarcoma AR = 54.7/10 K PY for all SMN

Fuchs, et al. (Mayo)	7.4 years	1975–1999	397	29	21 solid tumor 8 hematologic	NR	6.5% at 7.4 years	NR

Dunst, et al. (CESS)	5.1 years	1981–1991	674	8	3 solid tumor 5 hematologic	None significant, Trend toward increased solid SMN with any RT	0.7% 2.9% 4.7% NR	NR

Bacci, et al. (Rizzoli)	Range of 5–33 years	1972–1999	597	14	11 solid tumor 3 hematologic	Higher for “full dose” RT versus post-op “reduced dose”	3% 6.5% NR 12.7%	NR

Ginsberg, et al. (CCSS)	23 years (mean)	1970–1986	403	36	34 solid tumor 2 hematologic	NR	9% at 20 years	SIR = 5.9 AR = 48.1/10 K PY

Bhatia, et al. (CCG/POG)	8.03 years	1988-1992	587	16	4 solid tumor 12 hematologic	Treatment arm C	NR	SIR = 127.7 for myeloid SMN

Paulussen, et al. (EICESS)	4.6 years	1992-1999	690	6	2 solid tumor 4 hematologic	None significant, Trend toward increased risk after etoposide	0.93% NR NR NR	SIR = 30.9–33.4 for hematologic SMN (compared to Saarland & SEER registries)

Navid, et al. (SJCRH)	12.2 years	1979–2004	237	12	4 solid tumor 8 hematologic	More recent treatment protocols, Lower stage disease	3.0% 4.7% NR NR	SIR = 17.8 overall SIR = 65.1 on more recent protocols

Sultan, et al. (SEER)	6.7 years	1973–2005	1166	35	23 solid tumor 12 hematologic	Treatment era (1973–85), Any radiation (OR = 2.55)	2.1% 4.4% NR 8.0%	O/E = 4.01 O/E = 51.09 for myeloid SMN O/E = 51.08 for OS

Abbreviations: SMN: second malignant neoplasm, NCI: National Cancer Institute, SJCRH: St Jude Children's Research Hospital, CESS: Cooperative Ewing's Sarcoma Study, CCG: Children's Cancer Group, POG: Pediatric Oncology Group, EICESS: European Intergroup Cooperative Ewing's Sarcoma Study, SEER: Surveillance, Epidemiology and End Results, RT: radiation therapy, NR: not reported, AR: absolute risk, PY: person years, OR: odds ratio, SIR: standardized incidence ratio, O/E ratio of observed to expected cases OS: osteosarcoma
